# Clinical and diagnostic factors associated with 72-h unscheduled return to high-priority pediatric emergency care: a health services retrospective cohort study

**DOI:** 10.3389/fpubh.2026.1955991

**Published:** 2026-10-19

**Authors:** Jing Zhao, Lingli Pan, Qiu Jin, Jie Gao, Min Cheng

**Affiliations:** 1 Department of Emergency Nursing, West China Second University Hospital, Sichuan University, Chengdu, Sichuan, China; 2 Key Laboratory of Birth Defects and Related Diseases of Women and Children (Sichuan University), Ministry of Education, Chengdu, Sichuan, China

**Keywords:** 72-h unscheduled return, children, discharge transition, emergency care utilization, health services research, medical complexity, oncologic conditions, pediatric emergency care

## Abstract

**Background:**

Seventy-two-hour unscheduled return visits reflect short-term emergency care reutilization, discharge-transition safety, and disease progression in children. Evidence is limited for children managed through high-priority pediatric emergency care pathways, where triage acuity, observation needs, and disease-specific care processes differ from standard emergency department care. This study examined clinical and diagnostic factors associated with 72-h unscheduled return to such a pathway and assessed whether these associations differed between red-zone and yellow-zone encounters.

**Methods:**

We conducted a retrospective cohort study of 30,170 pediatric visits from 24,479 children managed through an institutionally defined high-priority pediatric emergency care pathway at a tertiary women’s and children’s hospital in China in 2025. The primary outcome was unscheduled return to the same pathway within 72 h after the index visit. Visits were ordered chronologically within each child. Mixed-effects logistic regression with a patient-level random intercept was used to account for repeated visits. Zone and triage level were not included together because they were structurally linked in the institutional workflow.

**Results:**

Overall, 1,507 index visits were followed by a 72-h return (5.0%). In the primary model, yellow-zone encounters had higher odds of return than red-zone encounters (adjusted odds ratio [aOR], 2.28; 95% confidence interval [CI], 1.34–3.87). Oncologic conditions showed the strongest association with return (aOR, 6.48; 95% CI, 3.54–11.87), followed by hematologic diseases (aOR, 2.49; 95% CI, 1.06–5.89). The zone-by-diagnosis interaction model indicated that the oncologic risk signal was amplified in the yellow zone (interaction aOR, 5.10; 95% CI, 1.25–20.84; *p* = 0.023). The oncologic association remained stable across sensitivity analyses.

**Conclusion:**

In this high-priority pediatric emergency care pathway, 72-h unscheduled return was more closely related to the underlying diagnostic category than to initial vital-sign abnormality. Yellow-zone children with oncologic conditions may require strengthened discharge assessment, explicit return precautions, and short-interval follow-up to improve care-transition safety and support risk-informed use of high-priority emergency resources.

## Introduction

Seventy-two-hour unscheduled return visits are commonly used as a short-term indicator of emergency care quality, discharge-transition safety, disease progression, and emergency care reutilization (1–5). In children, early return can be difficult to interpret because symptoms may evolve quickly, presentations may be nonspecific, and caregivers may seek repeated care when clinical changes are uncertain (6). Repeated emergency department use and frequent-visitor patterns may also shape short-term revisit risk and should be considered when interpreting pediatric emergency utilization data (7). Recent work has also characterized the demographic and clinical features of pediatric emergency department utilization in other settings (8). From a public health and health services perspective, identifying children at increased risk may support risk-informed discharge planning, care coordination, and more targeted use of high-priority emergency resources.

Most pediatric studies on 72-h return visits have been conducted in general emergency department populations or among children discharged from standard emergency care settings (1–5, 9, 10). Recent work has also examined revisits associated with serious or critical outcomes after emergency department discharge and found that clinically meaningful risk stratification matters for identifying high-risk children (9, 10). Children managed through high-priority pediatric emergency care pathways, however, may differ from these broader populations. They may have higher triage priority, acute physiologic instability, a need for close observation, or disease-specific care pathways. Findings from general pediatric emergency department cohorts may therefore not fully apply to this setting.

The term “resuscitation area” is not used uniformly across emergency care systems. In some settings, it refers narrowly to a room or unit for patients requiring immediate life-saving intervention. In our hospital, it denotes a broader, institutionally defined high-priority pediatric emergency care pathway. Children assigned triage levels I to III enter this pathway; levels I and II are managed in the red zone, and level III in the yellow zone. The cohort therefore includes not only children with critical physiologic instability, but also children who require rapid assessment, close monitoring, or special disease-related pathways.

Within this pathway, the yellow zone is usually less acutely unstable than the red zone at presentation, but it should not be assumed to be low risk. Yellow-zone encounters may include children with high-risk underlying conditions or evolving conditions that are not captured by a single set of vital signs.

Oncologic and hematologic diseases are particularly relevant in this context. Children with these conditions may present with fever, infection risk, treatment-related immunosuppression, bleeding risk, pain, gastrointestinal symptoms, or early deterioration that is initially nonspecific (11–14). Even when vital signs appear reassuring at the index visit, the underlying disease and treatment background may increase the likelihood of early re-presentation to high-priority emergency care. Few studies have specifically assessed whether oncologic or hematologic diagnoses are associated with short-term return to high-priority pediatric emergency care pathways.

A further question is whether return-related factors operate similarly across emergency zones. In many emergency department studies, return visits are analyzed as a single outcome across heterogeneous clinical areas. A zone-specific analysis may reveal subgroups who appear relatively stable during the index encounter but remain at increased risk after discharge or departure from the emergency care pathway.

This study therefore aimed to (1) identify clinical and diagnostic factors associated with 72-h unscheduled return to a high-priority pediatric emergency care pathway in a large pediatric emergency cohort, and (2) examine whether these associations differed between red-zone and yellow-zone encounters. We hypothesized that the underlying diagnostic category would be more strongly associated with return than vital-sign abnormality alone, and that risk patterns would vary by emergency zone. By focusing on short-term return to high-priority pediatric emergency care, this study provides health services evidence relevant to emergency resource use, discharge-transition safety, and follow-up planning for children with complex diagnostic backgrounds.

## Methods

### Study design and setting

This retrospective cohort study was conducted in the pediatric emergency department of a tertiary women’s and children’s hospital in China between January 1 and December 31, 2025. The analysis included visits managed through the hospital’s high-priority pediatric emergency care pathway.

In this hospital, the pediatric emergency resuscitation area functions as an institutionally defined high-priority care pathway rather than as a unit restricted to active cardiopulmonary resuscitation. Pediatric emergency triage is based on an institutionally developed four-level pediatric emergency triage criterion established using the Delphi method (15). Within the local workflow, children assigned triage levels I to III are managed in the high-priority pediatric emergency care pathway: levels I and II are allocated to the red zone, and level III is allocated to the yellow zone. Accordingly, the cohort should be interpreted as a high-priority pediatric emergency cohort managed through this pathway, not as a cohort limited to children requiring immediate resuscitation.

Although level III is the formal triage level assigned to the yellow zone in this pathway, 123 triage level-IV encounters were recorded in the yellow zone during the study period, even though level IV is not routinely assigned to the high-priority pediatric emergency care pathway. These encounters were retained in the primary analysis and excluded in a sensitivity analysis.

A new emergency registration and nurse triage are generated only for a new presentation to the emergency department. Repeat registration and triage are not generated during the same continuous episode of care, including emergency observation. Thus, a subsequent record for the same child indicates a new emergency presentation after leaving the hospital. The dataset did not capture lower-acuity revisits directed to other emergency or outpatient areas.

### Study population

All pediatric visits to the high-priority pediatric emergency care pathway during the study period were eligible. We included visits with a valid encounter identifier, patient registration number, registration time, emergency zone, triage information, diagnostic category, sex, and 72-h return outcome. Visits resulting in in-hospital death were excluded from the analytic cohort. Repeated visits by the same child were retained, and within-child correlation was addressed using patient-level random effects.

### Outcome definition

The primary outcome was unscheduled return to the high-priority pediatric emergency care pathway within 72 h after the index visit. For each child, visits during the study period were ordered chronologically by registration time, and every visit was evaluated as a potential index visit using a rolling 72-h window. An index visit was classified as followed by a 72-h unscheduled return if the next visit by the same child to the same pathway occurred within 72 h of the index registration time. A new emergency registration and nurse triage are generated only for a new presentation to the emergency department, not during the same continuous episode of care (including emergency observation); therefore, a subsequent record for the same child indicates a new presentation after leaving the hospital.

This definition identifies whether an index visit was followed by a return within 72 h; it does not classify the current visit as being a return from a previous encounter. In the primary analysis, every visit was treated as a potential index visit, including visits that were themselves returns, so that a return visit could be followed by a further return within 72 h (chained returns). Returns to other emergency or outpatient areas were not captured. Visits near the end of the study period that lacked a complete 72-h observation window were retained in the primary analysis and excluded in a sensitivity analysis.

### Variables

Candidate predictors were sex, age group, emergency zone, triage level, focused diagnostic category, abnormal vital-sign count, night-shift presentation, and season. Age-group boundaries were defined such that the lower bound was inclusive and the upper bound exclusive; therefore, each exact boundary value was assigned to the older age group (e.g., age 3 months was included in the 3 to <12 months group).

Emergency zone was classified as red or yellow. Triage level was the nurse-assigned category recorded at presentation. Because zone allocation was structurally linked to triage level in the institutional workflow, zone and triage level were not entered together in the primary model.

Primary diagnoses were collapsed into focused diagnostic categories based on the first diagnosis recorded in the hospital information system (HIS) diagnostic description set at the index visit: oncologic conditions, hematologic diseases, circulatory diseases, neonatal/perinatal diseases, and other diseases. When multiple diagnoses were recorded, the first was used, and categories were defined by keyword-based grouping aligned with ICD-11 chapters. In this hospital, pediatric oncologic diagnoses are established through hematology-oncology outpatient or inpatient care and require histopathological confirmation; the emergency department does not make a new cancer diagnosis. An oncologic diagnostic category recorded at an emergency visit therefore reflects a pre-existing cancer diagnosis under active follow-up. Neonatal status was defined according to age and the institutional triage policy. Because institutional policy routes all neonates through this pathway irrespective of diagnosis, the age-based neonate variable overlaps substantially with the neonatal/perinatal diagnostic category; this overlap is considered when interpreting neonatal and perinatal findings. This study did not measure a validated medical-complexity score. The focused diagnostic categories derived from the first recorded diagnosis were analyzed as diagnosis-based predictors and should not be interpreted as a formal measure of medical complexity.

Vital signs at presentation were used to calculate an abnormal vital-sign count according to predefined operational thresholds (Supplementary Table S1). The count (range 0–4) summed four binary indicators recorded at the index visit: fever (tympanic temperature ≥ 38.5 °C, the conventional threshold for antipyretic administration), hypoxia (oxygen saturation < 94%), tachycardia, and tachypnea, the latter two defined by age-specific thresholds. Missing vital-sign values were treated as absent (i.e., not counted as abnormal). Hypotension (systolic blood pressure < 90 mmHg, applicable only to children aged ≥ 5 years) was not included in the main count because blood pressure is not routinely measured in younger children. Night-shift presentation and season were derived from the visit time. Season was defined by meteorological seasons: season 1 = spring (March–May), season 2 = summer (June–August), season 3 = autumn (September–November), and season 4 = winter (December–February).

### Statistical analysis

Descriptive statistics were used to summarize cohort characteristics, the overall 72-h return rate, zone distribution, diagnostic distribution, and repeated-visit structure. Categorical variables are presented as counts and percentages. Continuous variables are presented as medians and interquartile ranges when appropriate. Characteristics were compared between visits followed by a 72-h return and those not followed by a return using chi-square or Fisher exact tests for categorical variables and nonparametric tests for continuous variables.

The primary analysis used mixed-effects logistic regression with a patient-level random intercept to account for repeated visits by the same child. The model included emergency zone, sex, age group, focused diagnostic category, abnormal vital-sign count, night-shift presentation, season, and the patient-level random intercept. Adjusted odds ratios (aORs) and 95% confidence intervals (CIs) were reported.

Because zone allocation was structurally linked to triage level, with levels I and II managed in the red zone and level III primarily managed in the yellow zone, emergency zone and triage level were not included together in the primary model. A sensitivity model replaced emergency zone with triage level to assess whether the main diagnostic findings were robust to an alternative adjustment for initial triage severity.

We conducted several sensitivity analyses. Because 123 triage level-IV encounters were recorded in the yellow zone even though level IV is not routinely assigned to the high-priority pediatric emergency care pathway, we repeated the primary model after excluding these encounters. Additional sensitivity analyses excluded neonates, restricted the analysis to each child’s first visit, excluded high-frequency patients, and restricted the cohort to encounters with a complete 72-h observation window. High-frequency patients were defined as children with five or more high-priority pediatric emergency care pathway visits during the study year. We also conducted a sensitivity analysis in which a visit that was itself a return could not serve as an index visit (i.e., only the first return following a non-return index visit was counted as the event), to assess whether chained returns influenced the estimates.

To assess zone-specific heterogeneity, we fitted an interaction model including emergency zone, focused diagnostic category, and their interaction term. Zone-specific comparisons of diagnostic categories were then derived from the interaction model.

All analyses were conducted using R. Visits with missing required analytic fields (*n* = 1,154; 3.7% of screened visits) were excluded during cohort construction, so that all regression models were fitted on complete cases. A two-sided *p*-value less than 0.05 was considered statistically significant. The manuscript was prepared with reference to STROBE reporting recommendations (16).

## Results

### Study cohort and 72-h return rate

A total of 31,357 visits were screened; 1,187 were excluded (1,151 for missing triage level or primary diagnostic data, 33 in-hospital deaths, 2 for a missing patient registration number, and 1 for missing sex), leaving 30,170 visits from 24,479 unique children managed through the high-priority pediatric emergency care pathway (Figure 1). Of these, 1,507 index visits were followed by an unscheduled return to the same pathway within 72 h, yielding an overall return rate of 5.0%. Among these 1,507 index visits, the corresponding return encounter was itself followed by a further return within 72 h in 401 cases (26.6%), constituting chained returns. Baseline characteristics by return status are shown in Table 1.

**Figure 1:**
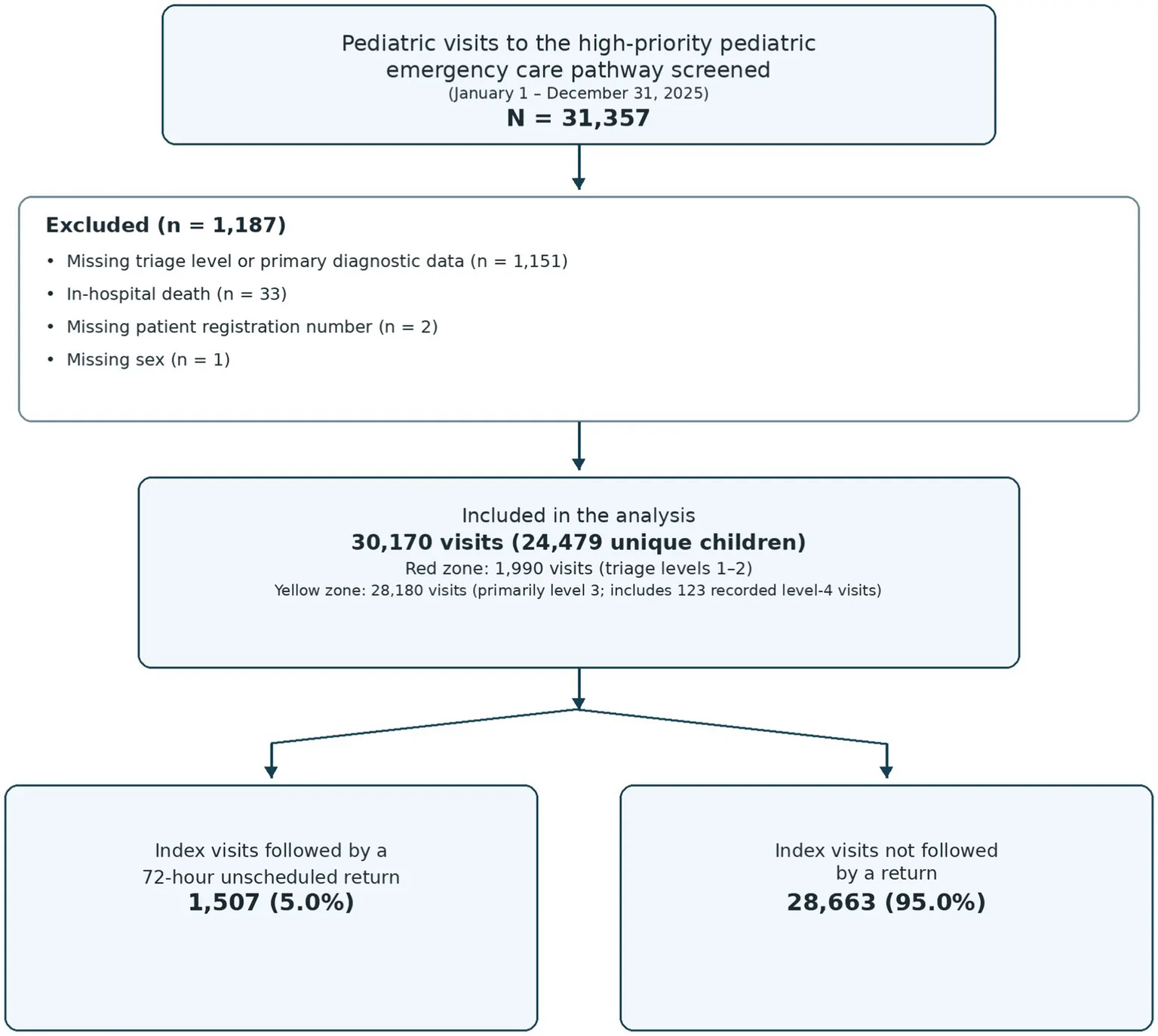
Flow diagram of the study cohort, including the screening and exclusion flow and the 72-h unscheduled return outcome definition.

**Table 1:** Baseline characteristics of pediatric visits according to 72-h unscheduled return status.

Characteristic	Level	No 72-h return	72-h return	*p*-value
Visits, *n*		28,663	1,507	
Sex	Male	16,294 (56.8)	962 (63.8)	<0.001
	Female	12,369 (43.2)	545 (36.2)	
Age, months	Median (IQR)	30.0 (5.6–79.0)	25.0 (0.9–84.0)	<0.001
Age group	<3 months	6,284 (21.9)	467 (31.0)	<0.001
	3 to <12 months	3,145 (11.0)	173 (11.5)	
	1 to <3 years	6,024 (21.0)	178 (11.8)	
	3 to <5 years	3,706 (12.9)	163 (10.8)	
	5 to <12 years	7,430 (25.9)	389 (25.8)	
	≥12 years	2074 (7.2)	137 (9.1)	
Neonate	Non-neonate	23,772 (82.9)	1,105 (73.3)	<0.001
	Neonate	4,891 (17.1)	402 (26.7)	
Emergency zone	Red zone	1951 (6.8)	39 (2.6)	<0.001
	Yellow zone	26,712 (93.2)	1,468 (97.4)	
Triage level	Level I	121 (0.4)	2 (0.1)	<0.001
	Level II	1830 (6.4)	37 (2.5)	
	Level III	26,601 (92.8)	1,456 (96.6)	
	Level IV	111 (0.4)	12 (0.8)	
Focused diagnostic category	Other diseases	21,988 (76.7)	636 (42.2)	<0.001
	Circulatory diseases	248 (0.9)	76 (5.0)	
	Oncologic conditions	1,540 (5.4)	365 (24.2)	
	Hematologic diseases	825 (2.9)	100 (6.6)	
	Neonatal/perinatal diseases	4,062 (14.2)	330 (21.9)	
Season	Spring (Mar–May)	8,181 (28.5)	422 (28.0)	<0.001
	Summer (Jun–Aug)	6,875 (24.0)	365 (24.2)	
	Autumn (Sep–Nov)	7,938 (27.7)	350 (23.2)	
	Winter (Dec–Feb)	5,669 (19.8)	370 (24.6)	
Night-shift presentation	No	24,745 (86.3)	1,364 (90.5)	<0.001
	Yes	3,918 (13.7)	143 (9.5)	
Abnormal vital-sign count	Median (IQR)	0.0 (0.0–0.0)	0.0 (0.0–0.0)	0.020

Values are presented as *n* (%) unless otherwise indicated. Continuous variables are presented as median (interquartile range). The outcome indicates whether the index visit was followed by an unscheduled return to the same high-priority pediatric emergency care pathway within 72 h. *p*-values were calculated using chi-square tests or Fisher’s exact tests for categorical variables and nonparametric tests for continuous variables, as appropriate.

### Crude return rates by emergency zone and diagnostic category

Crude 72-h return rates varied across emergency zones and diagnostic categories (Table 2 and Figure 2). The cohort included 1,990 red-zone visits and 28,180 yellow-zone visits. The crude return rate was 2.0% in the red zone and 5.2% in the yellow zone.

**Table 2:** Crude 72-h unscheduled return rates by emergency zone and focused diagnostic category.

Category	Level	Visits	Returns	Return rate, %
Overall	All visits	30,170	1,507	5
Emergency zone	Red zone	1990	39	1.96
	Yellow zone	28,180	1,468	5.21
Focused diagnostic category	Circulatory diseases	324	76	23.46
	Oncologic conditions	1905	365	19.16
	Hematologic diseases	925	100	10.81
	Neonatal/perinatal diseases	4,392	330	7.51
	Other diseases	22,624	636	2.81
Zone × focused diagnostic category	Red zone/Hematologic diseases	75	6	8
	Red zone/Oncologic conditions	89	6	6.74
	Red zone/Neonatal/perinatal diseases	155	3	1.94
	Red zone/Other diseases	1,564	24	1.53
	Red zone/Circulatory diseases	107	0	0
	Yellow zone/Circulatory diseases	217	76	35.02
	Yellow zone/Oncologic conditions	1816	359	19.77
	Yellow zone/Hematologic diseases	850	94	11.06
	Yellow zone/Neonatal/perinatal diseases	4,237	327	7.72
	Yellow zone/Other diseases	21,060	612	2.91

Crude return rates were calculated as the number of index visits followed by a 72-h unscheduled return divided by the total number of visits in each subgroup. Emergency zones were defined according to the institutional triage pathway: triage levels I–II were assigned to the red zone, and triage level III was assigned to the yellow zone. A total of 123 triage level-IV encounters were recorded in the yellow zone and were included in the primary analysis but excluded in a sensitivity analysis. Crude rates are unadjusted and should not be interpreted as independent associations.

**Figure 2:**
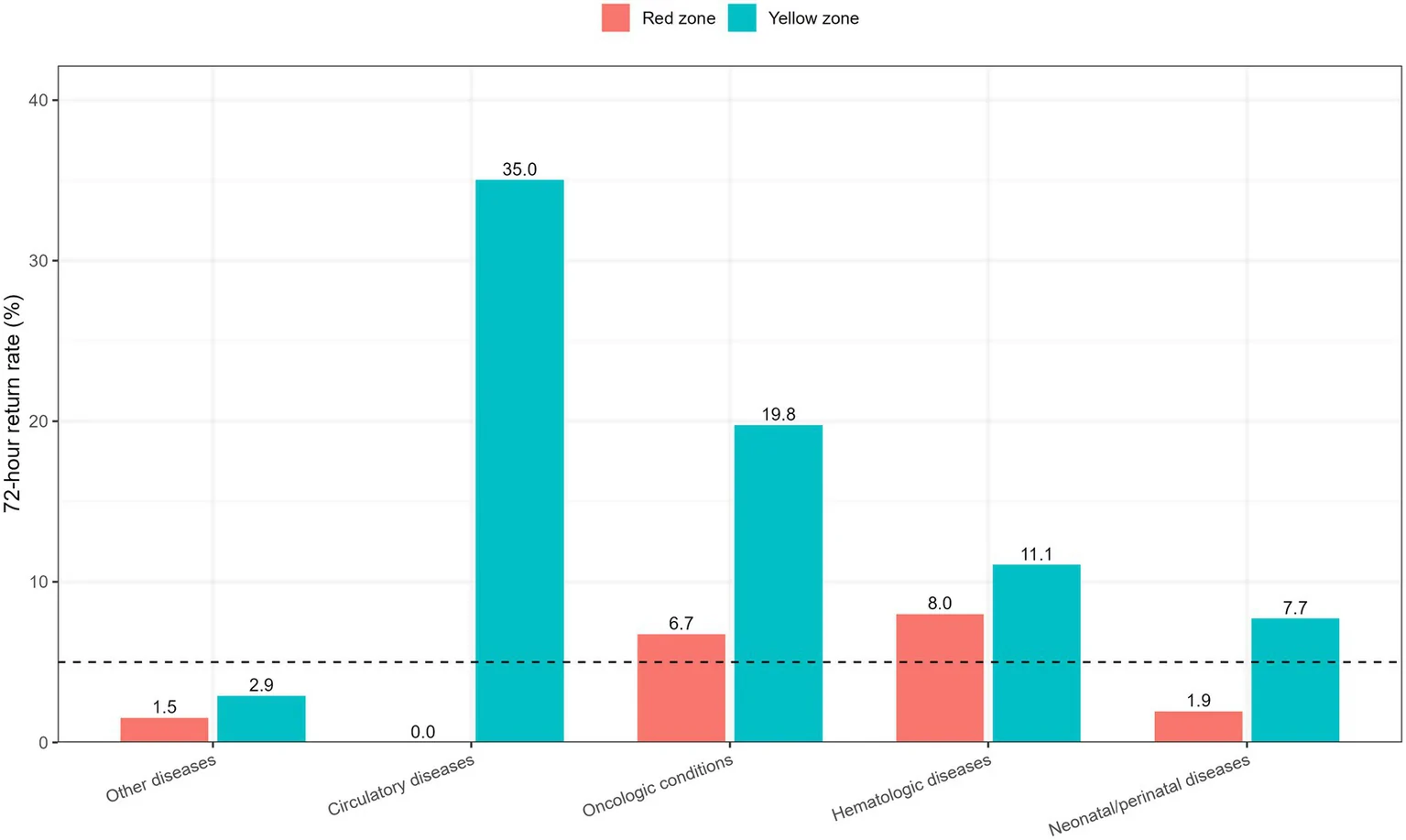
Crude 72-h unscheduled return rates by emergency zone and focused diagnostic category.

Oncologic conditions and hematologic diseases had higher crude return rates than most other diagnostic categories. Circulatory diseases also showed a high crude return rate, but this association did not persist after multivariable adjustment.

### Primary regression model

Because zone allocation was structurally linked to triage level in the institutional workflow, zone and triage level were not included together in the primary model. The primary mixed-effects logistic regression model included emergency zone, sex, age group, focused diagnostic category, abnormal vital-sign count, night-shift presentation, season, and a patient-level random intercept.

In the primary model, yellow-zone encounters had higher odds of 72-h unscheduled return than red-zone encounters (aOR, 2.28; 95% CI, 1.34–3.87). Oncologic conditions had the strongest diagnostic association with return (aOR, 6.48; 95% CI, 3.54–11.87), followed by hematologic diseases (aOR, 2.49; 95% CI, 1.06–5.89). Circulatory diseases, neonatal/perinatal diagnoses, abnormal vital-sign count, and night-shift presentation were not independently associated with return (Table 3 and Figure 3).

**Table 3:** Primary and sensitivity regression models for 72-h unscheduled return.

Variable	Primary model	Triage-level model	Excluding level-IV visits	Excluding neonates	First-visit analysis	Excluding high-frequency patients	Complete 72-h window	Excluding chained returns
Yellow zone vs. red zone	2.28 (1.34–3.87); *p* = 0.002		2.64 (1.53–4.55); *p* < 0.001	1.56 (0.93–2.61); *p* = 0.089	2.41 (1.57–3.90); *P* < 0.001	1.47 (0.69–3.13); *p* = 0.316	1.98 (1.18–3.32); *p* = 0.010	2.24 (1.10–4.57); *p* = 0.026
Triage level II vs. level I		0.15 (0.03–0.73); *p* = 0.019						
Triage level III vs. level I		0.38 (0.08–1.81); *p* = 0.223						
Triage level IV vs. level I		0.62 (0.09–4.39); *p* = 0.634						
Circulatory diseases vs. other diseases	1.39 (0.33–5.89); *p* = 0.658	1.39 (0.33–5.89); *p* = 0.659	1.18 (0.27–5.18); *p* = 0.827	0.83 (0.19–3.61); *p* = 0.801	1.33 (0.47–2.97); *p* = 0.533	1.28 (0.12–13.28); *p* = 0.836	0.67 (0.15–2.93); *p* = 0.592	1.62 (0.26–9.97); *p* = 0.604
Oncologic conditions vs. other diseases	6.48 (3.54–11.87); *p* < 0.001	6.09 (3.36–11.05); *P* < 0.001	6.14 (3.36–11.22); *P* < 0.001	6.13 (3.37–11.17); *P* < 0.001	8.57 (6.59–11.04); *P* < 0.001	3.62 (1.33–9.87); *p* = 0.012	6.26 (3.43–11.42); *P* < 0.001	5.76 (2.51–13.24); *P* < 0.001
Hematologic diseases vs. other diseases	2.49 (1.06–5.89); *p* = 0.037	2.23 (0.95–5.25); *p* = 0.066	2.67 (1.13–6.32); *p* = 0.025	2.63 (1.09–6.34); *p* = 0.031	3.95 (2.66–5.67); *p* < 0.001	1.85 (0.51–6.71); *p* = 0.347	2.59 (1.10–6.10); *p* = 0.029	1.94 (0.57–6.58); *p* = 0.289
Neonatal/perinatal diseases vs. other diseases	0.79 (0.49–1.27); *p* = 0.337	1.00 (0.62–1.61); *p* = 0.999	0.93 (0.58–1.50); *p* = 0.765	0.41 (0.07–2.24); *p* = 0.303	1.26 (0.97–1.65); *p* = 0.084	0.59 (0.34–1.02); *p* = 0.061	0.81 (0.50–1.30); *p* = 0.380	0.71 (0.35–1.42); *p* = 0.330
Abnormal vital-sign count	0.92 (0.77–1.09); *P* = 0.337	0.90 (0.76–1.07); *p* = 0.250	0.92 (0.77–1.09); *p* = 0.341	0.90 (0.76–1.08); *p* = 0.262	0.91 (0.79–1.04); *p* = 0.192	0.80 (0.60–1.05); *p* = 0.111	0.91 (0.76–1.08); *p* = 0.266	0.91 (0.71–1.16); *p* = 0.434
Night-shift presentation	0.95 (0.70–1.29); *p* = 0.727	0.95 (0.70–1.30); *p* = 0.755	0.97 (0.71–1.32); *p* = 0.836	0.89 (0.62–1.28); *p* = 0.534	1.02 (0.81–1.27); *p* = 0.871	1.07 (0.73–1.58); *p* = 0.731	0.93 (0.68–1.26); *p* = 0.622	0.66 (0.39–1.11); *p* = 0.116

Values are adjusted odds ratios with 95% confidence intervals and *p*-values. The primary model was a mixed-effects logistic regression model with a patient-level random intercept and included emergency zone, sex, age group, focused diagnostic category, abnormal vital-sign count, night-shift presentation, and season. Because emergency zone was structurally linked to triage level in the institutional workflow, emergency zone and triage level were not entered simultaneously in the primary model. The triage-level model replaced emergency zone with triage level. The level-IV sensitivity analysis excluded 123 encounters recorded in the yellow zone; level IV is not routinely assigned to the high-priority pediatric emergency care pathway. Additional sensitivity analyses excluded neonates, restricted the cohort to each child’s first visit, excluded high-frequency patients, restricted the analysis to visits with a complete 72-h observation window, and excluded chained returns. High-frequency patients were defined as children with five or more high-priority emergency care visits during the study year. The first-visit analysis used one visit per child and therefore did not require a patient-level random intercept. Blank cells indicate variables that were not included in the corresponding model or were not applicable.

**Figure 3:**
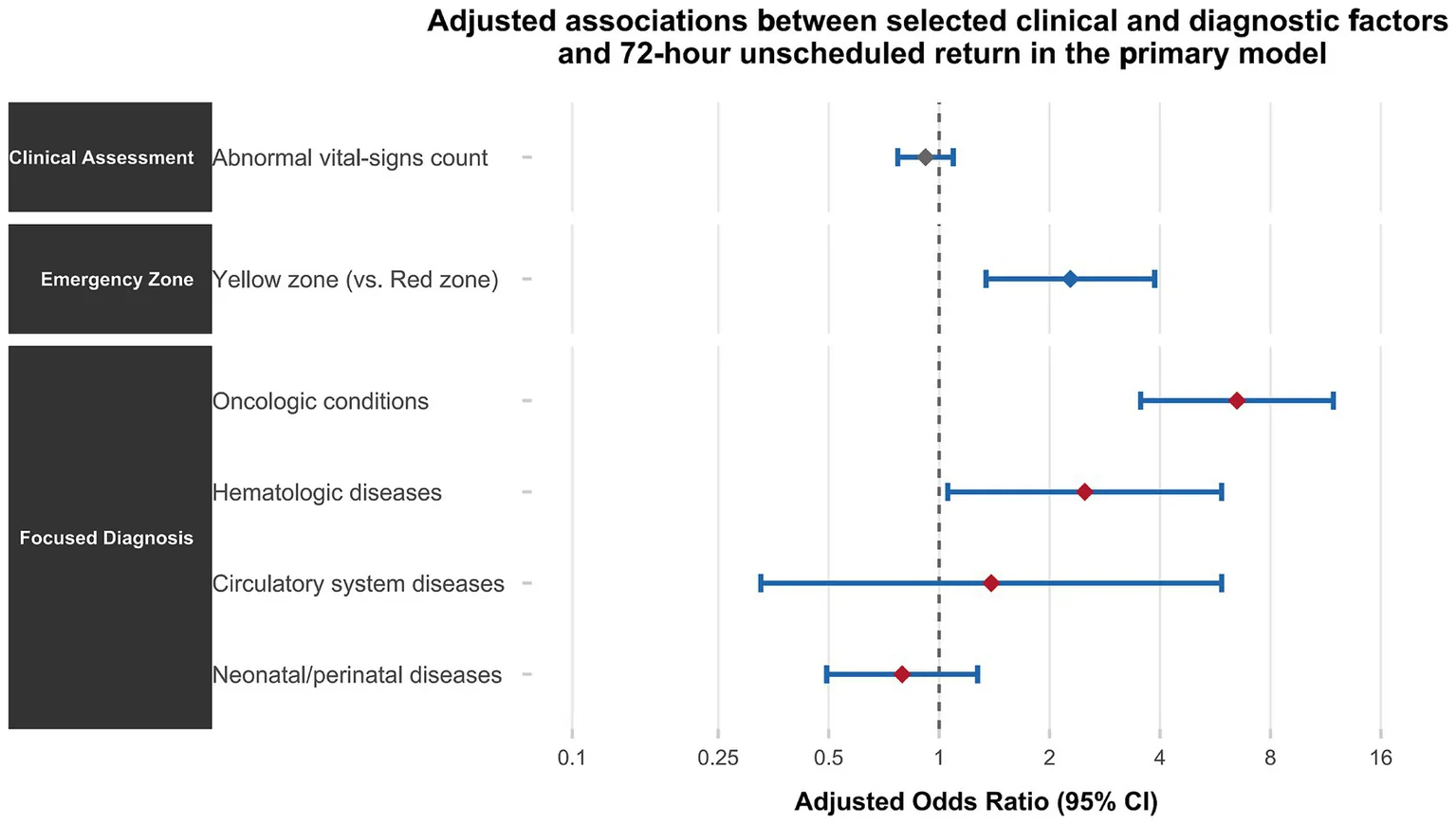
Adjusted associations between selected clinical and diagnostic factors and 72-h unscheduled return in the primary model.

### Sensitivity analyses

The association between oncologic conditions and 72-h return was stable across all sensitivity analyses, including analyses excluding neonates, restricting the cohort to each child’s first visit, excluding high-frequency patients, and restricting the cohort to encounters with a complete 72-h observation window. The corresponding sensitivity estimates are also shown in Table 3.

Hematologic diseases remained associated with higher return risk in most sensitivity analyses, but the association weakened after high-frequency patients were excluded. The yellow-zone association was also attenuated after excluding neonates and high-frequency patients, suggesting that part of the observed return risk in the yellow zone may reflect differences in patient composition, including the concentration of neonates and recurrent emergency users in this area.

Excluding the 123 triage level-IV encounters did not materially alter the primary estimates. The yellow-zone association remained consistent with the primary analysis (aOR, 2.64; 95% CI, 1.53–4.55), as did the associations for oncologic conditions (aOR, 6.14; 95% CI, 3.36–11.22) and hematologic diseases (aOR, 2.67; 95% CI, 1.13–6.32) (Table 3). Replacing emergency zone with triage level also did not materially change the interpretation of the main findings. In the sensitivity analysis in which a visit that was itself a return could not serve as an index visit (*N* = 28,663 visits; 1,106 returns), the oncologic association remained robust (aOR, 5.76; 95% CI, 2.51–13.24) and the yellow-zone association was essentially unchanged (aOR, 2.24; 95% CI, 1.10–4.57), whereas the hematologic association attenuated (aOR, 1.94; 95% CI, 0.57–6.58).

### Zone-specific heterogeneity

The zone-by-diagnosis interaction model suggested zone-specific heterogeneity for oncologic conditions (Table 4). The excess risk associated with oncologic conditions was amplified in the yellow zone, with a significant interaction between yellow-zone presentation and oncologic diagnosis.

**Table 4:** Zone-by-diagnosis interaction model and zone-specific diagnostic comparisons.

Section	Variable	OR (95% CI) or estimate	*p*-value
Interaction term	Yellow zone × circulatory diseases	Unstable estimate (sparse data)	0.925
	Yellow zone × oncologic conditions	5.10 (1.25–20.84)	0.023
	Yellow zone × hematologic diseases	0.35 (0.08–1.57)	0.172
	Yellow zone × neonatal/perinatal diseases	1.56 (0.19–12.63)	0.675
Zone-specific comparison	Red zone: Circulatory diseases vs. other diseases	Not reliably estimable (0 return events)	0.928
	Red zone: Oncologic conditions vs. other diseases	1.22 (0.27–5.46)	0.798
	Red zone: Hematologic diseases vs. other diseases	6.93 (1.32–36.29)	0.022
	Red zone: Neonatal/perinatal diseases vs. other diseases	0.52 (0.07–4.21)	0.544
	Yellow zone: Circulatory diseases vs. other diseases	1.70 (0.40–7.23)	0.475
	Yellow zone: Oncologic conditions vs. other diseases	6.20 (3.41–11.28)	<0.001
	Yellow zone: Hematologic diseases vs. other diseases	2.45 (1.04–5.77)	0.040
	Yellow zone: Neonatal/perinatal diseases vs. other diseases	0.82 (0.51–1.32)	0.415

The interaction model included emergency zone, focused diagnostic category, their interaction term, and the same covariates as the primary model. Interaction terms estimate whether the association between focused diagnostic category and 72-h unscheduled return differed between the red and yellow zones. Zone-specific comparisons were derived from the interaction model to aid clinical interpretation. The red-zone circulatory comparison was not reliably estimable because no 72-h return events occurred among 107 red-zone circulatory-disease visits; all other estimable zone-specific comparisons are presented with 95% confidence intervals. Estimates based on sparse events should be interpreted cautiously. OR, odds ratio; CI, confidence interval.

Zone-specific comparisons showed that oncologic conditions were strongly associated with 72-h return within the yellow zone, whereas no clear oncologic association was observed within the red zone. Hematologic diseases were associated with higher return risk within both zones, but without clear evidence of zone-specific effect modification. Interaction estimates for circulatory diseases in the red zone were unstable, likely because of sparse events.

## Discussion

### Main findings

In this cohort of children managed through an institutionally defined high-priority pediatric emergency care pathway, 5.0% of index visits were followed by an unscheduled return to the same pathway within 72 h. The most consistent finding was the strong association between oncologic conditions and return. This association remained stable across sensitivity analyses, including those excluding neonates, first-visit-only analyses, exclusion of high-frequency patients, and restriction to encounters with a complete 72-h observation window. Hematologic diseases were also associated with increased return risk in the primary model and in several sensitivity analyses, although the association weakened after high-frequency patients were excluded. In contrast, abnormal vital-sign count and night-shift presentation were not independently associated with return. These findings position short-term return as both a clinical outcome and a marker of pediatric emergency care utilization and care-transition risk. The observed 5.0% return rate is consistent with the range reported in previous pediatric return-visit studies (1–5), supporting the comparability of this cohort with general pediatric emergency populations despite the high-priority setting.

### Interpretation of oncologic conditions

The association between oncologic conditions and 72-h return makes clinical sense. Children with oncologic diseases often have complex treatment backgrounds, including chemotherapy-related immunosuppression, infection risk, hematologic complications, pain, gastrointestinal symptoms, and nonspecific early signs of deterioration (11–14). Vital signs at a single emergency visit may not capture these risks. Some children appear stable at initial assessment but remain vulnerable to rapid clinical change after leaving the emergency department. An oncologic diagnosis may therefore reflect clinical fragility and short-term uncertainty, not just acute severity at presentation. In our setting, an oncologic diagnosis is never first established in the emergency department; pediatric cancer requires histopathological confirmation and is diagnosed through hematology-oncology outpatient or inpatient care. Accordingly, all 1,905 oncologic encounters in the analytic cohort represented patients with a pre-existing cancer diagnosis under active follow-up. By contrast, the 925 hematologic encounters were more heterogeneous and may have included both chronic conditions under active follow-up and conditions newly diagnosed at the index visit; the available dataset did not permit a reliable numerical distinction between these groups.

### Zone-specific findings and yellow-zone oncologic patients

The zone-specific results are clinically important. Yellow-zone encounters had higher odds of 72-h return in the primary model, and the interaction model indicated that the oncologic risk signal was amplified in the yellow zone. In zone-specific comparisons, oncologic conditions were strongly associated with return within the yellow zone, whereas no clear oncologic association was observed within the red zone. This pattern identifies yellow-zone oncology patients as an important subgroup for short-term risk recognition. Although they may not appear critically unstable at the index visit, their underlying disease may increase the likelihood of early return to high-priority emergency care. However, the wide confidence interval for the interaction term (aOR 5.10, 95% CI 1.25–20.84) indicates limited precision, and this finding should be interpreted as exploratory until corroborated in larger samples.

### Interpretation of the yellow zone

The yellow zone should not be interpreted as uniformly low risk. In this institutional workflow, yellow-zone patients were generally less acutely unstable than red-zone patients at presentation, but the zone also included children with complex diagnostic backgrounds, neonates managed through the high-priority pediatric emergency care pathway by institutional policy, and children with repeated emergency care use. Recent evidence also suggests that neonatal emergency department utilization may vary across patient populations and healthcare contexts (17). The attenuation of the yellow-zone association after excluding neonates and high-frequency patients suggests that the observed return risk may partly reflect patient composition rather than zone assignment itself. This interpretation is consistent with recent evidence showing that children with medical complexity have high emergency care utilization and that both clinical complexity and broader contextual factors contribute to subsequent emergency department use (18, 19).

### Hematologic diseases

Hematologic diseases were associated with higher return risk in the primary model and in several sensitivity analyses, but the association weakened after high-frequency patients were excluded. This pattern suggests that part of the hematologic association may be related to recurrent emergency care use among children with chronic hematologic conditions. These patients may repeatedly require emergency assessment for fever, anemia, bleeding, pain, transfusion-related issues, or disease-related complications. Future studies should distinguish clinically unavoidable revisits from potentially preventable returns that may be reduced through structured discharge planning, early follow-up, or specialty coordination.

### Circulatory diseases

Circulatory diseases had a high crude return rate but were not independently associated with return after adjustment. This gap between crude and adjusted findings shows why multivariable adjustment matters in return-visit research. Crude diagnostic rates may be shaped by age distribution, zone allocation, repeated visits by the same child, sparse events in some subgroups, or clustering of specific high-risk patients. Crude return rates should therefore not be interpreted as independent diagnostic effects.

### Vital signs and night-shift presentation

The absence of an independent association between abnormal vital-sign count and 72-h return should be interpreted in context. In a high-priority pediatric emergency pathway, marked physiologic abnormality at the index visit may trigger more intensive assessment, treatment, observation, or admission, which may reduce early return among the most acutely unstable patients. Conversely, children with complex chronic conditions may have reassuring vital signs at presentation but remain vulnerable after discharge or departure from the pathway. This may explain why diagnostic background, particularly oncologic disease, was more consistently associated with return than vital-sign abnormality alone.

Night-shift presentation was not independently associated with 72-h return. In this dataset, short-term return appeared to be driven more by patient- and disease-level factors than by time of presentation. This result should not be read as evidence that crowding, staffing, or workflow variation has no role, because these factors were not directly measured. Previous studies have reported mixed findings regarding night-shift presentation: some have found an association with return visits (5), while others have not (9, 10). Our results align with the latter group and suggest that the relationship between shift timing and return depends on local workflow, staffing models, and case mix.

### Public health and clinical implications

The findings have practical implications for pediatric emergency care and health service planning. Children with oncologic conditions, especially those managed in the yellow zone, may benefit from more deliberate discharge assessment, explicit return precautions, and early follow-up arrangements. For children with complex diagnostic backgrounds, discharge communication should also account for caregiver understanding, care coordination needs, and family context, which have been identified as important factors in discharge instruction comprehension and adherence among children with medical complexity (20). Clinicians and health service managers should also recognize that physiologic stability during a single encounter does not necessarily indicate low short-term risk in children with complex chronic disease. Return-visit surveillance in high-priority pediatric emergency pathways may help identify groups that require targeted care pathways, particularly oncology and hematology patients. Risk stratification for pediatric emergency return should therefore incorporate the underlying diagnostic category alongside triage level and vital signs, supporting more efficient allocation of follow-up and high-priority emergency resources.

### Strengths

The cohort was large, comprising over 30,000 visits from nearly 25,000 unique children, which allowed multivariable modeling and subgroup analyses. Mixed-effects logistic regression with a patient-level random intercept accounted for within-child correlation arising from repeated visits. The zone-by-diagnosis interaction analysis identified zone-specific risk patterns that would have been obscured in a pooled model. Multiple sensitivity analyses (excluding neonates, restricting to first visits, excluding high-frequency patients, restricting to visits with a complete 72-h window) consistently supported the oncologic association.

### Limitations

Several limitations should be considered. First, this was a single-center study, and the local triage and zone-allocation policy may limit generalizability. The high-priority pediatric emergency care pathway was defined by the institutional workflow: triage levels I to III were managed in this pathway, with levels I and II assigned to the red zone and level III to the yellow zone. This structure may differ from hospitals where resuscitation areas are restricted to patients requiring immediate life-saving interventions. Still, the institutional triage framework shares key features with the Emergency Severity Index and the Paediatric Early Warning Score systems, including the use of tiered acuity levels to direct resource allocation, which may support partial comparability with settings that use similar structured triage approaches.

Second, the outcome captured only returns to the high-priority pediatric emergency care pathway; lower-acuity revisits to other emergency or outpatient areas were not included. The findings should therefore be interpreted as factors associated with return to high-priority pediatric emergency care, rather than to all pediatric emergency revisits. Third, all neonates were managed in the high-priority pediatric emergency care pathway by institutional policy, so neonatal and perinatal findings should be interpreted in this context. Fourth, data on final disposition, outpatient follow-up, caregiver decision-making, and preventability of return were incomplete, limiting the ability to distinguish disease progression, planned reassessment, and potentially preventable revisits. Fifth, a child with a known oncologic condition presenting for an unrelated reason could be classified according to the presenting diagnosis. Sixth, whereas oncologic diagnoses by definition reflect pre-existing cancer diagnoses under active follow-up (cancer is not first diagnosed in the emergency department), hematologic diseases are more heterogeneous and may include both chronic conditions under follow-up and conditions newly diagnosed at the index visit; the dataset does not reliably distinguish these, which may affect the interpretation of the hematologic association. Finally, although the mixed-effects model accounted for repeated visits by the same child, residual confounding by disease severity, treatment stage, or comorbidity burden may remain.

### Future directions

Future studies should validate these findings in multi-center cohorts, particularly in hospitals with different triage and zone-allocation policies, to assess generalizability. Prospective designs incorporating caregiver interviews, discharge instruction quality, and post-discharge follow-up data could help distinguish potentially preventable returns from those driven by unavoidable disease progression. The cost-effectiveness of targeted discharge protocols for yellow-zone oncology patients also warrants investigation. Future studies should also distinguish pre-existing diagnoses under active follow-up from new diagnoses made at the index visit, particularly for hematologic diseases.

## Conclusion

72-h unscheduled return to a high-priority pediatric emergency care pathway was more closely tied to the underlying diagnostic category than to initial vital-sign abnormality or night-shift presentation. Oncologic conditions showed the strongest association with return, particularly among yellow-zone encounters. Children with oncologic diseases who appear stable at the index visit may still need strengthened discharge assessment, clear return precautions, and short-interval follow-up. Adding the underlying diagnostic category to pediatric emergency return–risk recognition could support safer care transitions and more targeted use of high-priority emergency resources.

## Data Availability

The raw data supporting the conclusions of this article will be made available by the authors, without undue reservation.
