# Supplementary material for: Clinical and diagnostic factors associated with 72-h unscheduled return to high-priority pediatric emergency care: a health services retrospective cohort study
Source: Front Public Health. 2026 Oct 19;14:1955991. doi: 10.3389/fpubh.2026.1955991 (PMC13724413; doi:10.3389/fpubh.2026.1955991)
Supplement: Supplementary file 1 [file Table_1.docx]

**Supplementary Table S1. Age-specific thresholds used to define abnormal vital signs.**

| **Age group** | **Tachycardia (beats/min)** | **Tachypnea (breaths/min)** |
| --- | --- | --- |
| <3 months | > 180 | > 60 |
| 3 to <12 months | > 170 | > 50 |
| 1 to <3 years | > 160 | > 40 |
| 3 to <5 years | > 140 | > 35 |
| 5 to <12 years | > 130 | > 25 |
| ≥ 12 years | > 110 | > 20 |

*Fever: tympanic temperature ≥ 38.5 °C (the conventional threshold for antipyretic administration); hypoxia: oxygen saturation < 94%. Hypotension (systolic blood pressure < 90 mmHg, applicable only to children aged ≥ 5 years) was not included in the abnormal vital-sign count. Missing vital-sign values were treated as absent.* Age-group boundaries were defined such that the lower bound was inclusive and the upper bound exclusive; therefore, each exact boundary value was assigned to the older age group (e.g., age 3 months was included in the 3 to <12 months group).
